# Tracking of dendritic cell migration into lymph nodes using molecular imaging with sodium iodide symporter and enhanced firefly luciferase genes

**DOI:** 10.1038/srep09865

**Published:** 2015-05-14

**Authors:** Ho Won Lee, Seung Yun Yoon, Thoudam Debraj Singh, Yoon Ju Choi, Hong Je Lee, Ji Young Park, Shin Young Jeong, Sang-Woo Lee, Jeoung-Hee Ha, Byeong-Cheol Ahn, Yong Hyun Jeon, Jaetae Lee

**Affiliations:** 1 Department of Nuclear Medicine; 2 Department of Pharmacology; 3 Department of Pathology, School of Medicine, Kyungpook National UniversityDaegu; 4 Department of Nuclear Medicine, Dongnam Institution of Radiological & Medical SciencesBusan; 5 Leading-edge Research Center for Drug Discovery and Development for Diabetes and Metabolic Disease, Kyungpook National University Hospital, 807 Hogukro, Bukgu, Daegu; 6 Daegu-Gyeongbuk Medical Innovation Foundation (DGMIF), 80 Cheombok-ro, Dong-gu, Daegu, 701-310, Republic of Korea

## Abstract

We sought to evaluate the feasibility of molecular imaging using the human sodium iodide symporter (hNIS) gene as a reporter, in addition to the enhanced firefly luciferase (effluc) gene, for tracking dendritic cell (DCs) migration in living mice. A murine dendritic cell line (DC2.4) co-expressing hNIS and effluc genes (DC/NF) was established. For the DC-tracking study, mice received either parental DCs or DC/NF cells in the left or right footpad, respectively, and combined I-124 PET/CT and bioluminescence imaging (BLI) were performed. *In vivo* PET/CT imaging with I-124 revealed higher activity of the radiotracer in the draining popliteal lymph nodes (DPLN) of the DC/NF injection site at day 1 than DC injection site (*p* < 0.05). The uptake value further increased at day 4 (*p* < 0.005). BLI also demonstrated migration of DC/NF cells to the DPLNs at day 1 post-injection, and signals at the DPLNs were much higher at day 4. These data support the feasibility of hNIS reporter gene imaging in the tracking of DC migration to lymphoid organs in living mice. DCs expressing the NIS reporter gene could be a useful tool to optimize various strategies of cell-based immunotherapy.

Dendritic cells (DCs) are essential antigen-presenting cells (APCs) that stimulate helper and killer T cells *in vivo*^3^. Because DCs highly express MHC class I/II molecules, co-stimulatory molecules (B7), and adhesion molecules (ICAM-1, ICAM-3, and LFA-3), they can stimulate T-cells (CD4 and CD8 T-cells). Due to their unique characteristics, dendritic cells are able to identify various tumor antigens, such as AFP, CEA, and HPV-16 E6/E7, as well as undefined tumor antigens, which results in the induction of an immune response against tumor antigens in the host^4^. Although immunotherapeutic strategies with DCs have been applied to the treatment of diverse cancer models, their outcomes are only partially effective due to the inability to monitor the behavior of DCs *in vivo*. Thus, to be truly effective and practical, noninvasive imaging tools are needed to evaluate the efficacy of new DC-based immunotherapies that can accurately track the migration of DCs to lymphoid organs.

Several imaging techniques for imaging DC migration have been reported, including gamma scintigraphy with In-111^5^, positron emission tomography (PET) with F-18^6^, magnetic resonance imaging (MRI) using iron oxide magnetic nanoparticles^8^, near-infrared nanoparticles^9^, and reporter gene-based imaging^1^. Optical imaging methods, such as fluorescence (FLI) or bioluminescence imaging (BLI), have high sensitivity with low background, which enables tracking of the early distribution of infused DCs. However, these techniques cannot be applied to large animals due to depth limitations. MRI has been successfully applied to visualize the migration of DCs and provide fast imaging with high spatial resolution. But, it should require lots of contrast agents and there exist artifacts because of inaccurate coil sensitivities. Nuclear medicine imaging with direct or indirect labeling provide high sensitivity without depth limitation, and allows pharmacodynamics (PD)/pharmacokinetics (PK) study because of its ability of quantitative analysis. But, it has low spatial resolution and concern about radiation safety^4^.

Molecular imaging modalities with PET and SPECT have routinely been employed within clinical nuclear medicine and have been applied to various cells including immune cells^5^. The noninvasive imaging methods to visualize cells with these techniques can be divided as following; direct labeling and indirect labeling with reporter-mediated uptake of radionuclide. Direct labeling of cells is a rapid and easy procedure but it cannot show the cellular proliferation. In contrast, reporter gene-mediated imaging has the potential to faithfully visualize the viable cells longitudinally.

The most existing molecular-genetic imaging strategies are based on a reporter gene and a complementary reporter probe, which are transfected into cells to track them in living subjects^6^. The human sodium iodide symporter (hNIS) gene has been widely investigated to visualize gene expression, stem cells, and immune cells in pre-clinical and clinical models^7^. *In vivo* imaging based on the sodium iodide symporter (NIS) gene can be achieved using known radiotracers, such as Tc-99 m pertechnetate and radioiodine (I-123 and I-124), and a nuclear medicine imaging system. Furthermore, the NIS reporter gene is non-immunogenic and has no known adverse effects on cell viability and function, and this imaging technique does not require complex probe synthesis. These advantages of the NIS gene led us to further investigate whether the gene could be used for DC tracking in living organisms.

In this study, we investigated the feasibility of molecular-genetic imaging using an NIS gene as a reporter for tracking of DC migration toward lymphoid organs in living mice. Because the success of single-modality imaging remains limited for the visualization of the entire DC migration process, a multimodal imaging strategy is required to overcome the drawbacks of the use of individual modalities^8^. The combination of individual imaging modalities might allow for the visualization of migrating DCs at different phases of the immune response by exploiting the best features of each modality. Hence, we further adopted an optical reporter gene encoding enhanced firefly luciferase (effluc) that has been widely investigated in cell trafficking studies, including those on cytotoxic T lymphocyte (CTL) tracking, stem cell tracking, and DC tracking. This optical reporter gene facilitates tracking of the initial distribution of these cells due to its high sensitivity^3^. Immortalized DC cell line (DC2.4)^4^ has been well characterized and many researchers have applied it to immunologic research area^9^. For DC tracking study, DC2.4 cells simultaneously expressing a dual reporter gene system were established and used to employ both optical and nuclear molecular imaging. Serial BLI and I-124 PET/CT imaging were performed to assess the initial distribution and subsequent migration of infused DCs toward draining lymph nodes in living mice.

## Results

### Expression of reporter gene expression in DC/NF cells

**Figure 1:**
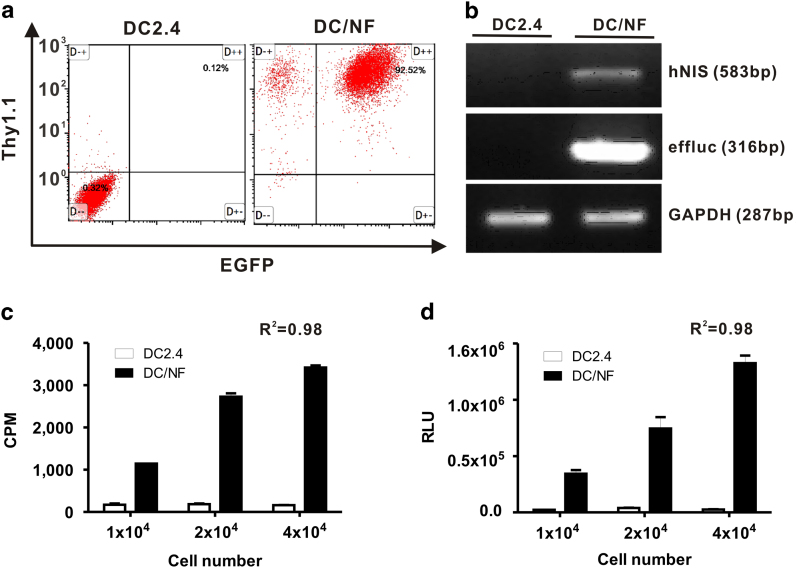
Establishment of dendritic cells co-expressing the hNIS and effluc genes. (**a**) Flow cytometric analysis of parental DC2.4 and DC/NF cells using an APC-Cy7-conjugated CD90.1 antibody. (**b**) RT-PCR analysis to determine the expression of the hNIS and effluc genes in parental DC2.4 and DC/NF cells. *Gapdh* was used as an internal control gene. (**c**) *In vitro* radioiodine uptake in DC2.4 and DC2.4/NF cells. (**d**) *In vitro* luciferase assay in parental DC2.4 and DC2.4/NF cells. Data are expressed as the mean ± standard deviation (SD) of 3 independent experiments.

As depicted in supplementary Fig. 1, DC2.4/NF cells were established by co-transfection with both 1) retrovirus co-expressing effluc and Thy1.1 genes and 2) retrovirus co-expressing effluc and Thy1.1 genes. Since Thy1.1 and EGFP genes are surrogate markers for effluc and NIS genes respectively, the population co-expressing Thy1.1 and EGFP genes could represent indirectly the percentage of NIS- and effluc- positive cells in stable transfectant. After labeling transfectant with Thy1.1-reactive antibody, the percentage of Thy1.1 + EGFP + cells was determined by flow cytometery. As illustrated in Fig. 1a, the hNIS and effluc genes were highly expressed in DC/NF cells, but not in parental DC2.4 cells. The expression levels of both genes in DC2.4 and DC/NF cells were 0.32% and 92.5%, respectively. Furthermore, to determine the gene expression of NIS and effluc genes in DC2.4/NF cells, RT-PCR analysis revealed two fragments with lengths of 583 bp and 316 bp for the hNIS and effluc genes in DC/NF cells, respectively, but not in parental DC2.4 cells (Fig. 1b).

### Assessment of functional activity for the hNIS and effluc gene

Since the expression of NIS protein in DC cells facilitate the influx of iodine in transfectant^7^, we assessed the functional activity of NIS reporter gene by radioiodine uptake assay. As shown in Fig. 1c, I-125 uptake increased in DC/NF cells, but not in parental DC2.4 cells, in a cell number-dependent manner. Radioiodine uptake was 21-fold higher in DC/NF cells than in parental DC2.4 cells (R^2^ = 0.98). The effluc protein in DC cells can react with D-luciferin as a substrate for effluc gene and finally emits light centered on 560 nm. Thus, we evaluated the functional activity of effluc using *in vivo* optical imaging system in live DC/NF cells after addition of D-luciferin to cells. BLI activity was approximately 3,000-fold higher in DC/NF cells than in parental DC2.4 cells, and BLI signals also increased in a cell number-dependent manner (Fig. 1d; R^2^ = 0.982).

### *In vivo* BLI and I-124 PET/CT imaging of DC/NF cells injected subcutaneously and intramuscularly to mice

**Figure 2:**
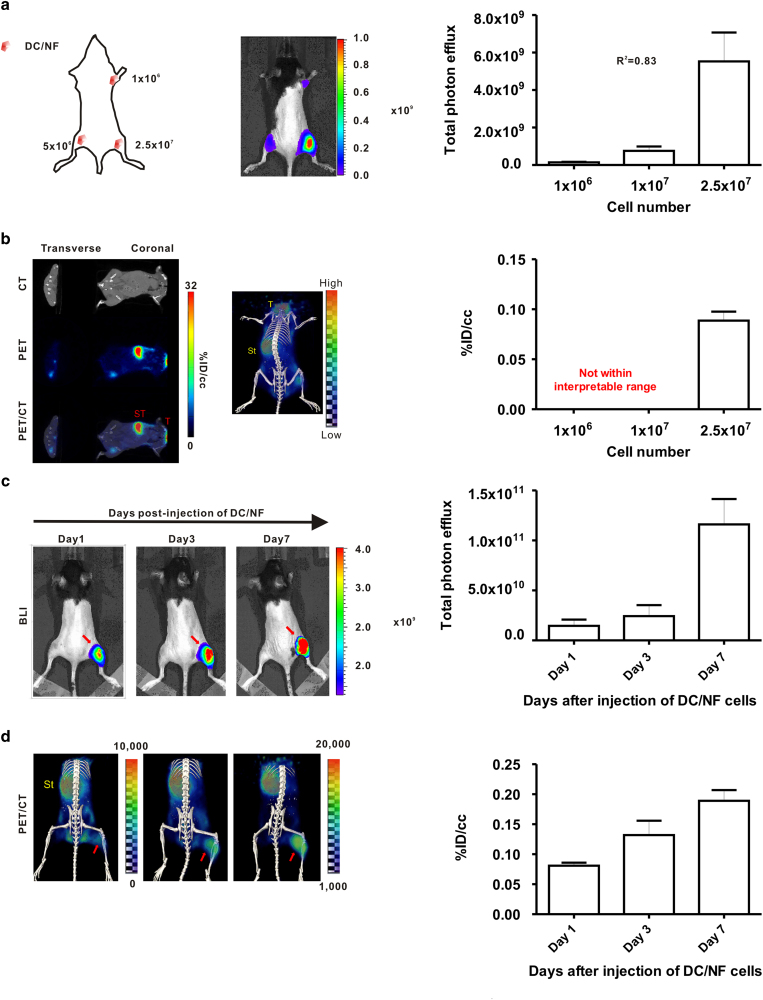
*In vivo* I-124 PET/CT imaging and BLI of DC2.4/NF cells in mice. DC/NF cells were intramuscularly administered in the right upper thigh (1 × 10^6^), left lower thigh (5 × 10^6^), and right thigh (2.5 × 10^7^) of mice, and imaging was acquired. (**a**) *In vivo* BLI of DC/NF cells in mice. (**b**) *In vivo* I-124 PET/CT imaging of DC/NF cells in living mice. Mice received DC/NF cells by intramuscular injection into the right thigh, and imaging was acquired at the indicated times. *In vivo* visualization of the proliferation of infused DC/NF cells with both (**c**) BLI and (**d**) I-124 PET/CT in living mice. Physiological iodide uptake was observed in the thyroid (T) and stomach (ST). Red arrows indicate the site injected with DC/NF cells. The uptake of radioiodine in the region of interest was evaluated with PMOD software and is expressed as %ID/cc (percent injected dose per cc). Data are expressed as the mean ± standard deviation (SD) of 3 independent experiments (n = 5 mice).

To evaluate the functional expression of both NIS and effluc genes in inoculated DC/NF cells by *in vivo* imaging, combined BLI and I-124 PET/CT imaging were performed after intramuscular injection with different numbers of DC/NF cells (Fig. 2a). *In vivo* BLI revealed evident signals in the DC/NF injection sites, and the signal intensity was correlated with an inoculated number of DC/NF cells (1 × 10^6^, 5 × 10^6^, and 2.5 × 10^7^ DC/NF cells, 1.4 × 10^8^ ± 2.7 × 10^7^, 7.6 × 10^8^ ± 2.4 × 10^8^, and 5.5 × 10^9^ ± 1.5 × 10^9^ P/cm^2^/s/sr, respectively; R^2^ = 0.83). Small animal PET/CT imaging with I-124 revealed focal uptake in the right hind thigh corresponding to an area of the highest number of inoculated cells (Fig. 2b, uptake of site injected with 2.5  × 10^7^ DC/NF cells: 0.0887 ± 0.009% ID/g) but not observed in sites injected with 1  × 10^7^ or 2.5 × 10^7^ DC/NF cells. As usual, the intense accumulation of I-124 was also observed in endogenously NIS expressing organs such as the thyroid and stomach.

The delivery of reporter gene into target cells by retroviral and lentiviral system leads to integration of gene of interest to chromosome of host, finally resulting in stable expression of exogenous genes, which enable to monitor the engrafted tumor, infused stem cell and immune cells in living subjects over time, noninvasively and repetitively^6^. We examined whether *in vivo* imaging with the NIS and effluc genes reasonably reflects the proliferation of DCs in living mice. As illustrated in Fig. 2c and 2d, focal uptake of I-124 as well as bright BLI signals were clearly seen in the DC/NF-injected site, but not in the parental DC2.4-injected site, at day 1 post-injection. Radioactivity measured from PET/CT images increased until day 7, and the uptake of the radiotracer within the DC/NF injection site was 2.3-fold higher at day 7 than that at day 1. A gradual increase in the BLI signal was also detected at the DC/NF injection site until day 7, but not at the parental DC2.4 injection site, and the BLI signal increased up to 7.9-fold that observed at day 1. These results indicate that *in vivo* imaging with combination of NIS and effluc genes can visualize the survival and proliferation of DC/NF cells in the host.

### The effect of viral infection on DC function

**Figure 3:**
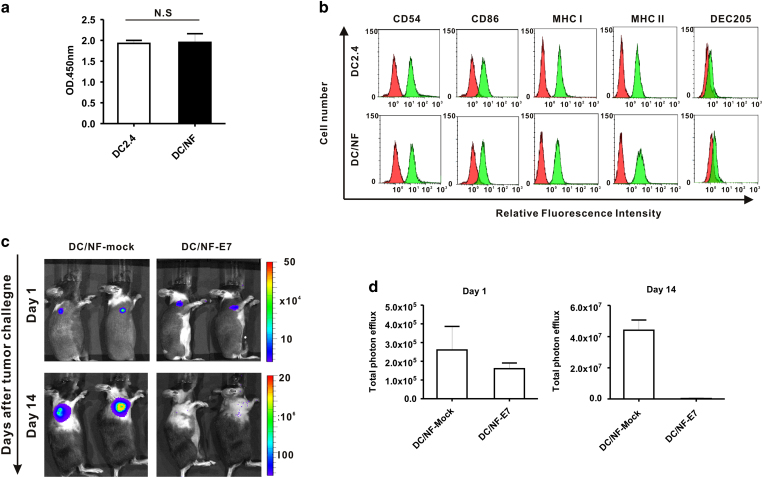
Effect of transduction of the NIS gene on DC function. (**a**) Cell proliferation rates in parental DC2.4 and DC/NF cells. There was no significant difference between the two cell lines. (**b**) Phenotypic analysis of DC2.4 and DC2.4/NF cells. Both DC2.4 and DC2.4/NF cells were stained with PE-conjugated CD54, CD86, H-2Kb (MHC Class I) and I-A/I-E (MHC class II), and APC-conjugated CD205 (DEC-205), respectively. Red histograms represent the isotype control. (**c**) *In vivo* BLI of tumor progression using the Rluc gene. (**d**) Quantification of the BLI signal emitted from the tumor lesion on days 1 and 14 post-tumor challenge. Marked inhibition of tumor formation is observed with immunization of E7-transfected DC/NF cells. Data are expressed as the mean ± standard deviation (SD) of 3 independent experiments.

Reporter genes have been applied to track metastasis of cancer, migration of immune cells or stem cells in living subjects^0^, but few reports have suggested possible adverse cellular effects. To address this issue, we investigated the proliferation ability and phenotype expression level between parental DC2.4 and DC/NF cells. The CCK assay indicated that there was no significant difference in cell proliferation between parental DC2.4 and DC/NF cells (Fig. 3a; not significant). Phenotype analysis revealed that the expression of DC-specific markers such as CD54, CD86, DEC-205, and MHC class I and II was also not different between parental DC and DC/NF cells (Fig. 3b).

Furthermore, DCs as professional antigen presenting cells (APCs) should induce the antitumor immunity through stimulation of effector cells like CD8 and CD4 T cells^1^. We examined whether the transfection of reporter genes could adversely affect tumor protection generated by immunization with DCs. The results of our *in vivo* study, performed to investigate whether immunization with DC/NF cells could generate protective effects against tumor formation in immunocompetent mice, are illustrated in Supplementary Fig. 2. There was no significant difference in the BLI activity of the Rluc gene between the two groups on day 1 after tumor challenge. However, tumor growth in mice immunized with E7-transfected DC/NF cells was significantly lower compared with mock-transfected DC/NF cells at day 14 after tumor challenge (Fig. 3c and d). These results demonstrated that the introduction of dual reporter gene does not affect cellular function of DC cells as well as generation of its anti-tumor immunity.

### *In vivo* imaging of DC migration to draining popliteal lymph nodes with combined I-124 PET/CT imaging and BLI

To visualize the migration of DC/NF cells toward DPLNs, both DCs were subcutaneously injected into the respective hind leg footpads as described in Supplementary Figure 4. The injection of immune cells can lead to non-specific uptake of radioiodine at injection site by immune cell-mediated inflammatory reaction. Due to these reasons, the DC2.4-injected footpad was further added as control site.

**Figure 4:**
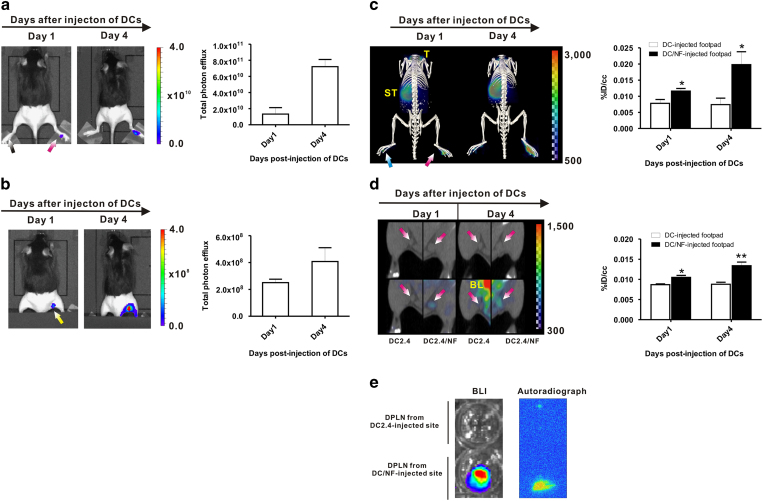
*In vivo* imaging of DC migration using the NIS and effluc genes in living mice. (**a**) *In vivo* BLI of DC/NF cells injected into footpad without masking the footpad signals (left panel). Quantification of BLI signals in the footpad (right panel). Red arrows indicate the footpad injected with DC/NF cells, and black arrows indicate the DC2.4-injected footpad. (**b**) *In vivo* BLI of DCs migrating into the DPLNs from DC/NF cells injected into the footpad with masking of the footpad for high activity (left panel). Quantification of BLI signals in the DPLNs (right panel).Yellow arrows indicate DPLNs from the DC-injected footpad. (**c**) 3D-reconstructed PET/CT imaging of DC/NF cells in the footpad (left panel). Quantification of radioiodine uptake in the respective footpads (right panel). (**d**) PET/CT imaging of DCs migrated into the DPLNs from the DC/NF-injected footpad (left panel). Quantification of radioiodine uptake in the respective DPLNs (right panel). (**e**) Representative *ex vivo* BLI and autoradiography in excised DPLNs. Physiological iodide uptake was observed in the thyroid (T), stomach (ST), and bladder (BL). The uptake of radioiodine in the region of interest was evaluated with PMOD software and is expressed as a %ID/cc (percent injected dose per cc). Data are expressed as the mean ± standard (SD) of 3 independent experiments. *, *p* < 0.05, **, *p* < 0.01.

BLI images were obtained on days 1 and 4 after injection of DCs. On day 1 post-injection of DCs, strong BLI signals were detected in the DC/NF-injected footpad, but not in the DC2.4-injected footpad. Signal intensity from the DC/NF-injected footpad further increased on day 4 (Fig. 4a). When the whole body was imaged without masking the DC-injected footpad, we could not trace DC migration towards the DPLNs, which may be due to strong signals arising from the DC/NF-injected footpad. Thus, *in vivo* BLI was performed after masking the footpad to allow detection of distinct signals in the DPLNs. Migrated DC/NF cells were clearly observed in the DPLNs from the DC/NF-injected footpad at day 1. BLI activity in the DPLNs from the DC/NF-injected footpad was much higher on day 4, revealing that the value of the BLI signal for the DPLNs from the DC/NF-injected footpad on day 1 and day 4 were 2.50 × 10^8^ ± 2.57 × 10^7^ and 4.07 × 10^8^ ± 1.03 × 10^8^ P/cm^2^/s/sr, respectively, (Fig. 4b).

In accordance with the results of BLI, intense uptake of radioiodine was detected in the DC2.4/NF-injected footpad but not in the DC2.4-injected footpad at day 1. The uptake values of the DC2.4-injected footpad and the DC/NF-injected footpad were 0.0078 ± 0.0008% ID/cc and 0.0116 ± 0.0012% ID/cc, respectively (Fig. 4c, *p* < 0.05 DC-injected footpad versus DC/NF-injected footpad). On day 4, the radioactive signals from the DC/NF-injected footpad were much higher than those of the DC2.4-injected footpad on day 1. The % ID/cc values of the DC/NF-injected footpad on day 4 was 0.0198 ± 0.004% ID/cc. However, there was no increase in radioiodine uptake in the DC2.4-injected footpad from day 1 to day 4. Interestingly, the radioiodine signal from DPLNs from the DC/NF-injected footpad was more intense than that from the DC2.4-injected footpad at day 1 after injection of DCs (*p* < 0.05), and accumulation of I-124 in the DPLNs from the DC/NF-injected footpad was found to have increased further at day 4 (Fig. 4d, *p* < 0.01). The radioiodine uptake of the DC/NF-injected footpad was 0.0105 ± 0.0005% ID/cc and 0.0134 ± 0.0009% ID/cc at day 1 and day 4, respectively. But, there were no significant differences in radioiodine uptake in the DPLNs from the DC2.4-injected footpad between day 1 and day 4.

Concurrently, *ex vivo* BLI revealed strong signals in DPLNs from the DC/NF-injected footpad but not in the DPLNs from the DC2.4-injected footpad (Fig. 4e, left panel). Autoradiography revealed that the radioactive signal was much higher in the DPLNs from the DC/NF-injected footpad than in the DPLNs from the DC2.4-injected footpad, which is consistent with the findings of *ex vivo* BLI (Fig. 4e, right panel). These results suggest that NIS gene is suitable reporter gene to track the DC migration noninvasively, like effluc gene, which has been already reported to be a valuable reporter for cell tracking.

### Immunohistological analysis in DPLNs

**Figure 5:**
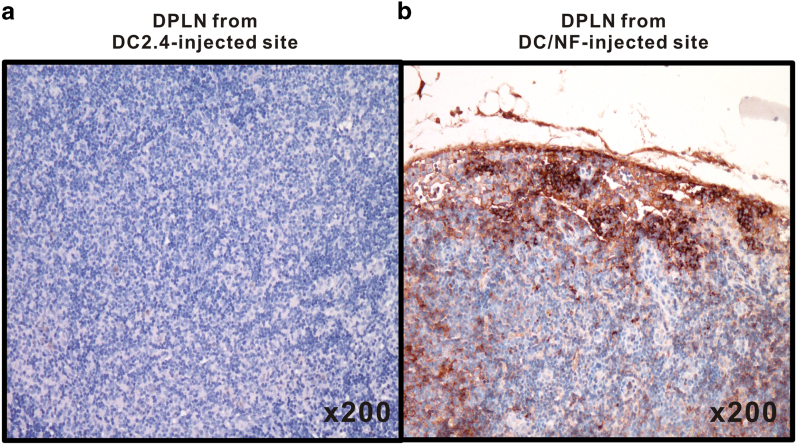
Immunohistological analysis of GFP expression in DPLNs. (**a**) DPLNs of DC-injected footpad, (**b**) DPLNs of DC/NF-injected footpad. Corresponding DPLNs from footpads injected with either parental DCs or DC/NF cells were excised after imaging, and immunohistochemical staining was performed using a GFP-specific antibody to determine the localization of DC/NF cells in the DPLNs. Arrows indicate GFP-positive cells in the DPLNs.

Because DC/NF cells express NIS, EGFP, effluc and Thy1.1 gens simultaneously, we selected the EGFP protein among four exogenous proteins to perform further immunuhistologcial analysis with EGFP-reactivity antibody. As shown in Fig. 5b, immunohistochemical staining of excised DPLNs taken from the DC/NF-injected footpad of the mice revealed that DC/NF cells is mainly located in subcapsular region and exhibited diffuse and strong positive reactivity for GFP. However, negative staining for GFP as a surrogate marker for the NIS gene, was observed in DPLNs from the DC2.4-injected footpad (Fig. 5a).

## Discussion

Tumor antigen-loaded DCs were recently reported to be used as a cancer vaccine to prevent tumors or eradicate existing tumors, encouraging many clinical trials evaluating the possibility of using DCs to generate an immune response against tumor-specific or tumor-associated antigens in lung cancer, melanoma, and colon cancer^2^. However, various concerns must be resolved before their clinical application. Among these concerns is the current lack of knowledge regarding cellular functionality following *in vitro* stimulation with tumor antigens, as well as the unknown mobility and fate of cells after adoptive transfer in the host. Noninvasive techniques of imaging of DC migration will be important for studying the role of DCs with an aim to develop effective cancer vaccines.

Molecular imaging with several reporter genes can be used to monitor the expression of the gene of interest (exogenous and endogenous) and several intracellular biological phenomena^2^. Most importantly, these methods allow researchers to non-invasively monitor the proliferation and migration of infused cells, which are important aspects of cell-based therapy in living subjects. Similar to the luciferase and HSV1-tk genes that are extensively investigated for their use in cell tracking, the NIS gene has also been demonstrated to be an attractive reporter gene for monitoring cancer progression and therapeutic response^6^, stem cell trafficking^8^, and specific cellular events^0^ in living subjects. Recently, several studies have demonstrated the possibility of using the NIS gene as a reporter for the tracking of T cells and macrophages. For example, Sharif-Paghaleh *et al.* demonstrated the distribution of adoptively transferred regulatory T cells in living mice with an NIS reporter gene and Tc-99 m SPECT/CT^1^. We have also reported the visualization of macrophage migration to inflamed tissues with the NIS gene and I-124 PET^2^ and subsequently reported dual reporter gene imaging strategies with the NIS and effluc genes to track macrophage migration and overcome the limitations of the NIS gene^3^. These sequential reports suggest that NIS gene transfection using a viral system does not adversely affect the function and viability of immune effector cells, and more importantly, *in vivo* imaging with both the NIS gene and nuclear medicine imaging devices is a feasible technique for noninvasively visualizing the migration of immune cells. To date, the NIS reporter gene has not been investigated for tracking dendritic cells in living subjects, although extensive research has been focused on molecular-genetic imaging with the NIS gene in various fields.

In this study, we demonstrated that nuclear medicine imaging with the NIS gene is a feasible technique for tracking the migration of DCs to lymphoid organs in living mice. Because *in vivo* imaging with the NIS gene is hampered by both high background noise and signals emitted from normal tissue and non-specific organs, it is difficult to visualize the initial distribution and low population of infused DCs. To overcome these hurdles during DC tracking with the NIS gene, *in vivo* nuclear medicine imaging was also combined with BLI based on the effluc gene, which allows the tracking of the early distribution of DCs and makes it easy to verify the feasibility of the NIS gene as a reporter gene for DC tracking. Thus, an effluc reporter gene was additionally adopted for DC tracking, and dual reporter DCs expressing both the NIS and effluc genes were successfully established with a viral vector system expressing the reporter genes. The expression of both NIS and effluc genes was confirmed by FACS analysis with respective surrogate markers (GFP and Thy1.1 as surrogate markers for NIS and effluc genes, respectively). Functional activity for the respective reporter genes was confirmed by determining the iodide uptake and by the luciferase assay, demonstrating that iodide uptake and luciferase activity is proportional to the cell number. When DC/NF cells were subcutaneously injected into mice, BLI signals were detected in the DC/NF-injected region in a cell number-dependent manner. Contrary to BLI, I-124 PET/CT imaging revealed focal uptake of radioiodine only in the inoculated site with the highest cell number due to the high background activity. However, I-124 PET/CT successfully demonstrated the proliferation of DC/NF cells in mice following intramuscular injection of DC/NF cells from day 1 to day 7.

We confirmed that cellular proliferation and the expression of phenotypic markers in transduced DC/NF cells was not altered by viral infection. We also examined whether DCs expressing dual reporter genes can generate antitumor effects equivalent to those generated by parental DCs in immunocompetent mice. To address these questions, cells from a murine cervical cancer cell line TC-1, expressing the HPV-16 E7 gene (cervical tumor-specific antigen), were employed as target cancer cells for DC-based immunotherapy, they are widely used in animal tumor models to investigate the therapeutic effects of DC vaccines expressing the E7 gene^6^. TC-1 cells were further transduced with a lentivirus co-expressing the Rluc gene and mCherry to monitor the tumor response in DC-based immunotherapy. In this study, to create a DC-based cancer vaccine capable of generating E7-specific antitumor immunity, DC/NF cells were transfected with an E7-encoding pcDNA/E7 vector. Finally, immunization with DC/NF cells transfected with an E7 antigen can successfully generate tumor protection in mice with cervical cancer. These results are consistent with those from a previous report by Wu *et al.* that demonstrated strong antitumor effects by immunization of E7-transfected DCs in a cervical cancer model^7^. These findings suggest that the transduction of the NIS gene does not affect DC function, as well as generating antitumor responses.

Several routes for DC injection, including the subcutaneous, intramuscular, intravenous, and intradermal routes, have been examined to optimize the protocol of DC-based immunotherapy in preclinical or clinical models^8^. To accomplish our *in vivo* tracking study, we selected the subcutaneous region of the footpad as a primary injection site for DCs, which has been commonly used for DC tracking imaging studies^9^. Animal PET/CT imaging on day 1 post-transfer revealed the accumulation of radioiodine in the DC/NF-injected footpad as well as a strong BLI signal at the same site. Signals from both PET/CT and BLI in the DC/NF-injected footpad further increased on day 4 post-injection compared with those from I-124 PET/CT and BLI taken at day 1. PET/CT imaging with I-124 as well as *in vivo* BLI demonstrated the migration of DCs to the DPLNs from the DC/NF-injected footpad at day 1, and both radioactivity and BLI signals emitted from the DPLNs also increased on day 4 compared with those on day 1. The localization of migrated DC/NF cells in the excised DPLNs was confirmed by *ex vivo* BLI and autoradiography. Our findings are consistent with those obtained from other studies that demonstrated the migration of DCs transferred via subcutaneous footpad using a nanoparticles^0^. Noh *et al.* have previously tracked migration of DCs to draining lymph nodes using NIR fluorescent nanoparticles following injection of labeled DCs in the footpad. The migration of the infused DCs was observed to draining lymph nodes from 12 h until 72 h post-transfer of labeled DCs, which is similar to that ofour study.

The approach using the NIS gene in this study was facilitated by the development of both I-124 PET/CT technology and quantitative analysis techniques based on CT imaging in small lesions^5^. Based on our current imaging strategy, we believe that this approach also could be used to track the migration of DCs administered through intramuscular and intravenous routes. Although this system is believed to be very useful in the optimization of DC-based immunotherapies, some issues remain to be addressed prior to application of DC tracking techniques in other disease models.

In conclusion, we successfully monitored the migration of DCs to lymphoid organs in living mice using nuclear medicine imaging of the NIS gene in conjunction with optical imaging of the effluc gene. More importantly, it should be noted that the migration of DCs into the DPLNs can be detected from early time points (in our case, from day 1 post-injection) using an NIS reporter gene by careful ROI analysis at DPLNs. Future studies should focus on tracking the migration of primary DCs using a new tracer for the NIS gene (for example, F-18 TFB) in living mice and on expanding these results to other immune cells such as CTLs and natural killer cells.

## Methods

### Ethics statement

All described procedures were reviewed and approved by Kyungpook National University (KNU-2012-43) Animal Care and Use Committee, and performed in accordance with the Guiding Principles for the Care and Use of Laboratory Animals.

### Animals

Pathogen-free six-week-old female C57bl/6 mice were purchased from SLC Inc. (Shizuoka, Japan).

### *In vivo* imaging

#### Study 1

1 × 10^6^, 5 × 10^6^, or 2.5 × 10^7^DC/NF cells were intramuscularly inoculated into the right fore, left hind, or right hind thigh of mice, respectively, and combined BLI and I-124 PET/CT imaging was performed (n = 5 mice).

#### Study 2

Ten million of either parental DCs or DC/NF cells were intramuscularly injected into the right hind thigh of mice, and BLI and I-124 PET/CT imaging was performed at the indicated times (n = 5 mice).

#### Study 3

Mice received either parental DCs or DC/NF cells by intramuscular injection once a week for 2 weeks. At one week after the last vaccination, TC-1/RM cells were injected into the right thigh of the hind legs of the mice, and sequential BLI imaging for the Rluc gene was performed to monitor tumor growth at the indicated times (n = 5 mice/group).

#### Study 4

To monitor migration of DCs toward lymphoid organs, animal experiments were conducted as depicted in Supplementary Figure 4. Parental DC2.4 and DC/NF cells (5 × 10^6^ DCs/mouse) were subcutaneously administered into the footpads of the left and right hind legs of mice, respectively (n = 6 mice), and BLI and I-124 PET/CT images were obtained on days 1 and 4 post-injection.

### Statistical analysis

All data are expressed as the mean ± standard deviation (SD) from at least three representative experiments, and statistical significance was determined using an unpaired Student’s test. p-values of <0.05 were considered statistically significant.

## Author Contributions

H.W.L. contributed to acquiring data, analyzing data and drafting the manuscript. S.Y.Y. and T.D.S. contributed to conception and design for this study. Y.J.C., H.J.L. and J.Y.P. contributed to enhancing intellectual content for this study. S.Y.J., S.W.L., J.H.H. and B.C.A. contributed to revising the manuscript J.T.L. and Y.H.J. contributed the conception, design, analyzing data, revising the manuscript, and approving the final content of the manuscript. All authors discussed the results and commented on the manuscript.

## Additional Information

**How to cite this article**: Lee, H. W. *et al.* Tracking of dendritic cell migration into lymph nodes using molecular imaging with sodium iodide symporter and enhanced firefly luciferase genes. *Sci. Rep.* **5**, 9865; doi: 10.1038/srep09865 (2015).

## Supplementary Material

Supplementary Information
