# Corrigendum: Tracking of dendritic cell migration into lymph nodes using molecular imaging with sodium iodide symporter and enhanced firefly luciferase genes

**DOI:** 10.1038/srep14397

**Published:** 2015-10-09

**Authors:** Ho Won Lee, Seung Yun Yoon, Thoudam Debraj Singh, Yoon Ju Choi, Hong Je Lee, Ji Young Park, Shin Young Jeong, Sang-Woo Lee, Jeoung-Hee Ha, Byeong-Cheol Ahn, Yong Hyun Jeon, Jaetae Lee

Scientific Reports 5: Article number: 986510.1038/srep09865; published online: 05142015; updated: 10092015

This Article contains errors in the Acknowledgements section:

“This work was supported by a National Research Foundation of Korea (NRF) grant funded by the Korean government (MEST, 2009-0078234); the National Nuclear R&D Program through the National Research Foundation of Korea (NRF) funded by the Ministry of Education, Science and Technology (No. 2012M2A2A7014020); a grant from the Korea Health Technology R&D Project, Ministry of Health & Welfare, Republic of Korea (A111345); grant no. RTI04-01-01 from the Regional Technology Innovation Program of the Ministry of Knowledge Economy (MKE) and the Kyungpook National University Research Fund (2013); a grant from the Medical Cluster R&D Support Project of Daegu Gyeongbuk Medical Innovation Foundation, Republic of Korea and by the National Research Foundation of Korea (NRF) Grant funded by the Korean Government (MSIP)(2014R1A1A1003323); a grant from Korea Health Technology R&D Project through the Korea Health Industry Development Institute (KHDI), funded by the Ministry of Health & Welfare, Republic of Korea (HI15C0001).”

should read:

“This work was supported by National Research Foundation of Korea(NRF) grant funded by the Korea government(MSIP) (No.2009-0078222, 2009-0078234), a grant of the Korea Health Technology R&D Project, Ministry of Health & Welfare (HI11C1300), National Nuclear R&D Program through the National Research Foundation of Korea(NRF) funded by the Ministry of Education, Science and Technology (No.2012M2A2A7014020), a grant from the Medical Cluster R&D Support Project through the Daegu-Gyengbuk Medical Innovation Foundation(DGMIF), funded by the Ministry of Health & Welfare (grant number:HT13C0002), BK21 PlusKNU Biomedical Convergence Program, Department of Biomedical Science, Kyungpook National University, National Research Foundation of Korea(NRF) Grant funded by the Korea Government(MSIP) (No.2014R1A1A1003323), a grant of the Korea Health Technology R&D Project through the Korea Health Industry Development Institute (KHIDI), funded by the Ministry of Health & Welfare (grant number : HI15C0001), and the National Research Foundation of Korea(NRF) grant funded by the Korea government(MSIP) (No. NRF-2015M2A2A7A01045177). Radioactive sodium iodide (Na124I) was provided by MC-50 cyclotron in Molecular Imaging Research Center, KIRAMS (2015038444).”

