# Supplementary material for: Tracking of dendritic cell migration into lymph nodes using molecular imaging with sodium iodide symporter and enhanced firefly luciferase genes
Source: Sci Rep. 2015 May 14;5:9865. doi: 10.1038/srep09865 (PMC4431315; doi:10.1038/srep09865)

Supplementary file

Tracking of dendritic cell migration into lymph nodes using molecular imaging with sodium iodide symporter and enhanced firefly luciferase genes

Ho Won Lee[1], Young Hyun Jeon[1], Seung Yun Yoon[1], ThoudamDebraj Singh[1], Yoon Ju Choi[1], Hong Je Lee[4], Ji Young Park[3], Shin Young Jeong[1], Sang-Woo Lee[1], Jeoung-Hee Ha[2],Byeong-Cheol Ahn[1], and Jaetae Lee[1]

[1]Department of Nuclear Medicine, [2]Department of Pharmacology, [3]Department of Pathology, School of Medicine, Kyungpook National University, Daegu and [4]Department of Nuclear Medicine, Dongnam Institution of Radiological & Medical Sciences, Busan, Republic of Korea,

Correspondence

Jaetae Lee, M.D., Ph.D.

Department of Nuclear Medicine, School of medicine, Kyungpook National University Hospital

50 Samduk 2-ga, Daegu 700-721

Republic of Korea

Tel.: 82-53-420-5586

Fax: 82-53-422-0864

E-mail: jaetae@knu.ac.kr

Yong Hyun Jeon, Ph.D.,

Leading-edge Research Center for Drug Discovery and Development for Diabetes and Metabolic Disease, Kyungpook National University Hospital,

807 Hogukro, Bukgu, Daegu 702-210

Republic of Korea

H.P: 82-10-2455-6046

Work: 82-53-200-3148, 82-53-200-3149

E-mail: jeon9014@gmail.com

Supplementary Methods

Ethics statement

All described procedures were reviewed and approved by Kyungpook National University (KNU-2012-43) Animal Care and Use Committee, and performed in accordance with the Guiding Principles for the Care and Use of Laboratory Animals.

Reagents

All antibodies were purchased from BD Biosciences (San Jose, CA, USA).

Cell lines

The murine dendritic cell line DC2.4 was cultured in RPMI1640 medium supplemented with 10% fetal calf serum (Hyclone, Logan, UT), 0.05 mM β-mercaptoethanol (Sigma-Aldrich, St. Louis, MO), 1% non-essential amino acids (Gibco, Grand Island, NY), and 1% penicillin-streptomycin (Gibco) at 37℃ in a 5% CO_2 atmosphere. DC2.4 cells were retrovirally transduced to express both the effluc and Thy1.1 genes. Thy1.1-positive cells were sorted using CD90.1 microbeads (Miltenyi Biotec, Bergisch Gladbach, Germany). DC2.4 cells expressing the effluc gene were further retrovirally transduced to express both the hNIS and enhanced green fluorescent protein (EGFP) genes. After staining the cells with anAPC-Cy7-conjugated Thy1.1 antibody (BD Biosciences), the Thy1.1+ EGFP+ population was enriched using FACS Aria III (BD Biosciences). The established stable cell line co-expressing effluc and hNIS is referred to as the DC/NF cell.

The murine cervical cancer cell line TC-1 was kindly provided by Dr. T. C. Wu. TC-1 cells were transduced with a lentivirus co-expressing the Renilla luciferase (Rluc) and mCherry genes (Genecopoeia Inc., Rockville, MD). Two days later, mCherry-positive cells were enriched using the FACS Aria III. The BLI activity of the Rluc gene in TC1-1/RM cells but not in parental TC-1 cells increased in a cell-number dependent manner (Supplementary Figure 3A). The established stable clone co-expressing Rluc and mCherry genes is referred to as the TC-1/RM cell line.

RT-PCR analysis

Parental DC2.4 and DC/NF cells were lysed using TRIzol solution (Invitrogen, Carlsbad, CA), and the total RNA was extracted according to the manufacturer's instructions. Reverse transcription was performed using a Revert-Aid First Strand cDNA Synthesis kit (Fermentas, Ontario, CA). In brief, 2 µg total RNA were reverse-transcribed in a final volume of 20 µlof reaction mixture containing 1 µloligo (dT) primer, 4 µl 5X reaction buffer, 2 µl 10mM dNTP mix, and 1 µl MuLV reverse transcriptase (Fermentas, Burlington, Ontario, CA). The hNIS gene was amplified with forward (5′-CGCTGGCCCAGAACCATC-3′) and reverse (5′-AAAATCTAGAGTCAGAGGTTTGTCTCCTGCT-3′) primers and 2 units of Taq DNA polymerase (Takara, Shiga, Japan) using a GeneAmp PCR system (Bio-Rad, Hercules, CA). After denaturation of the samples for 1 min at 94℃, the temperature profile was as follows: 30 cycles of 25 s at 94℃, 30 s at 57℃, and 30 s at 72℃,with an additional 10 min at 72℃. The effluc gene was amplified with forward (5′-GCACAAGGCCATGAAGAGAT-3′) and reverse (5′-CTTCTTGCTCACGAACACCA-3′) primers, with the same amplification conditions as for hNIS. Samples were resolved by electrophoresis in an ethidium bromide-stained agarose gel.

DC2.4/NF cells were transfected with either pcDNA/mock or pcDNA/E7 using X-tremeGENE9 transfection reagent (Roche, Indianapolis, IN) according to the manufacturer's instructions. The pcDNA/E7 vector was a gift from Dr. T.C. Wu (Johns Hopkins University). Two days after transfection, the transfectants were lysed using a TRIzol solution (Invitrogen, Carlsbad, CA), and the total RNA was extracted according to the manufacturer's instructions. Reverse transcription was performed using a Revert-Aid First Strand cDNA Synthesis kit (Fermentas, Ontario, CA). In brief, 2 µg total RNA were reverse-transcribed in a final volume of 20 µl containing 1 µloligo(dT) primer, 4 µl 5X reaction buffer, 2 µl 10 mM dNTP mix, and 1 µl MuLV reverse transcriptase (Fermentas, Burlington, Ontario, CA). The E7 gene was amplified with the 5′ and 3′ E7 primers and 2 units of Taq DNA polymerase (Takara, Shiga, Japan) using a Gene Amp PCR system (Bio-Rad, Hercules, CA, USA). After denaturation of the samples for 4 min at 94℃, 35 cycles of 1 min at 94℃, 1 min at 50℃, and 2 min at 72℃ were followed with an additional 10 min at 72 ℃. Samples were resolved by electrophoresis in an ethidium bromide-stained agarose gel. The gene expression of the E7 gene in E7-transfected DC/NF cells was determined by RT-PCR analysis (Supplementary Figure 3B).

Reporter gene assay

For luciferase assays, the cells were plated in white and clear-bottom 96-well plates. The appropriate substrate was added to each well (D-luciferin for effluc gene and coelenterazine h for Rluc gene) 24 h later, and BLI signals were measured using a microplate reader (BMG LabTech, Offenburg, Germany).

For the I-125 uptake assay, the indicated number of cells was plated in 24-well plates. One day after seeding, the cells were incubated with 500 µl of Hank's balanced salt solution (HBSS) containing 0.5% bovine serum albumin (bHBSS), 3.7 kBq carrier-free I-125, and 10µmol/L sodium iodide (specific activity 740 MBq/mmol) at 37℃ for 30 min. After incubation, the cells were washed twice as quickly as possible with ice-cold bHBSS buffer and lysed using 500 µl of 2% SDS. The radioactivity was measured using a Packard Cobra II gamma-counter (PerkinElmer, Waltham, MA).

Cell proliferation assay

Cell proliferation was determined using a Cell Counting Kit (CCK)-8 (Dojindo Laboratories, Tokyo, Japan). To examine cell proliferation, parental DC2.4 cells and DC/NF cells were plated at 2×10^4 cells per well in 96-well plates. Two days later, 20 µl CCK-8 solution was added to each well, and plates were incubated at 37℃ for 3 h. The absorbance was measured at 450 nm using a BMG LabTech microplate reader.

Phenotypic analysis

Parental DC2.4 and DC/NF cells were stained with PE-conjugated CD54, CD86, H-2Kb (MHC Class I), and I-A/I-E (MHC class II), and APC-conjugated CD205 (DEC-205) at 4℃ for 30 min. Cells were washed twice with 0.1% BSA/PBS and analyzed using the FACS AriaIII flow cytometer. Isotype-matched monoclonal antibodies were used as controls.

In vivo imaging

BLI was performed 10 min after either an intraperitoneal injection of D-luciferin (3 mg/mouse; PerkinElmer) or coelenterazineh (15 µg/mouse, NanoLight Technology, Pinetop, AZ) using the IVIS Lumina II

imaging system (PerkinElmer). Grayscale photographic images and bioluminescent color images were superimposed using LIVINGIMAGE (version 2.12, PerkinElmer) and IGOR Image Analysis FX software (WaveMetrics, Lake Oswego, OR). BLI signals were expressed in units of photons per cm^2 per second per steradian ($P/cm^2/s/sr$). After BLI imaging, 5.55 MBq I-124 was administered to the mice intravenously, and a 20-min scan was performed with the Triumph II PET/CT system (LabPET8, Gamma Medica-Ideas, Waukesha, WI) at 4 h post-injection. The PET imaging system has the following characteristics: ring diameter, 162 mm; FOV, 60 mm; crystals, 3072; spatial resolution, 1.35 mm FWHM FOV; and noise-equivalent counts, 37 kcps at 245 MBq (250–650 keV). CT scans were performed with an X-ray detector (fly acquisition; number of projection: 512; binning setting: 2×2; frame number: 1; X-ray tube voltage: 75kVp; focal spot size: 50 μm; magnification factor: 1.5; matrix size: 512) immediately following the acquisition of PET images. PET images were reconstructed by 3D-OSEM iterative image reconstruction, and the CT images reconstructed using filtered back-projections. All mice were anesthetized using 1-2% isoflurane gas during imaging. PET images were co-registered with anatomical CT images using 3D image visualization and analysis software VIVID (Gamma Medica-Ideas, Northridge, CA). To measure the uptake (%ID/cc) for the volumes of interest (VOIs), the VOIs from each image were manually segmented from co-registered CT images using both VIVID and PMOD software (PMOD Technologies, Zurich, Switzerland), and the uptake in the region of interest was measured with PMOD 3.5 software.

Ex vivo BLI imaging and autoradiography

After imaging, the mice were sacrificed, and the draining popliteal lymph nodes (DPLNs) were excised. Excised DPLNs were placed in white and clear-bottom 96 well-plates, and *ex vivo* BLI was acquired. Subsequently, excised DPLNs were exposed to imaging plates overnight, and the film was developed using a BAS-2000 (Fujifilm FLA-2000, Fujifilm, Japan) for autoradiography.

Immunohistology

Excised DPLNs were preserved in 10% formalin until assayed. At assay, the preserved DPLNs were embedded in paraffin and sectioned to 5-μm thin sections. For immunohistological analysis of GFP expression,

the paraffin sections were incubated for 1 h with an anti-GFP antibody (Millipore, Bedford, MA) at 1:100. Sections were then incubated for 30 min with a goat anti-rabbit antibody conjugated to peroxidase (Immunotech Laboratories, Monrovia, CA).

Supplementary Figures

Supplementary Figure 1 Schematic representation of DC/NF cells. DC/NF cells were derived from DC2.4 cells, a murine dendritic cell line, and stably express four genes; NIS for I-124 PET/CT imaging, effluc gene for in vivo BLI imaging, EGFP and thy1.1 for surrogate of NIS and effluc genes, respectively.

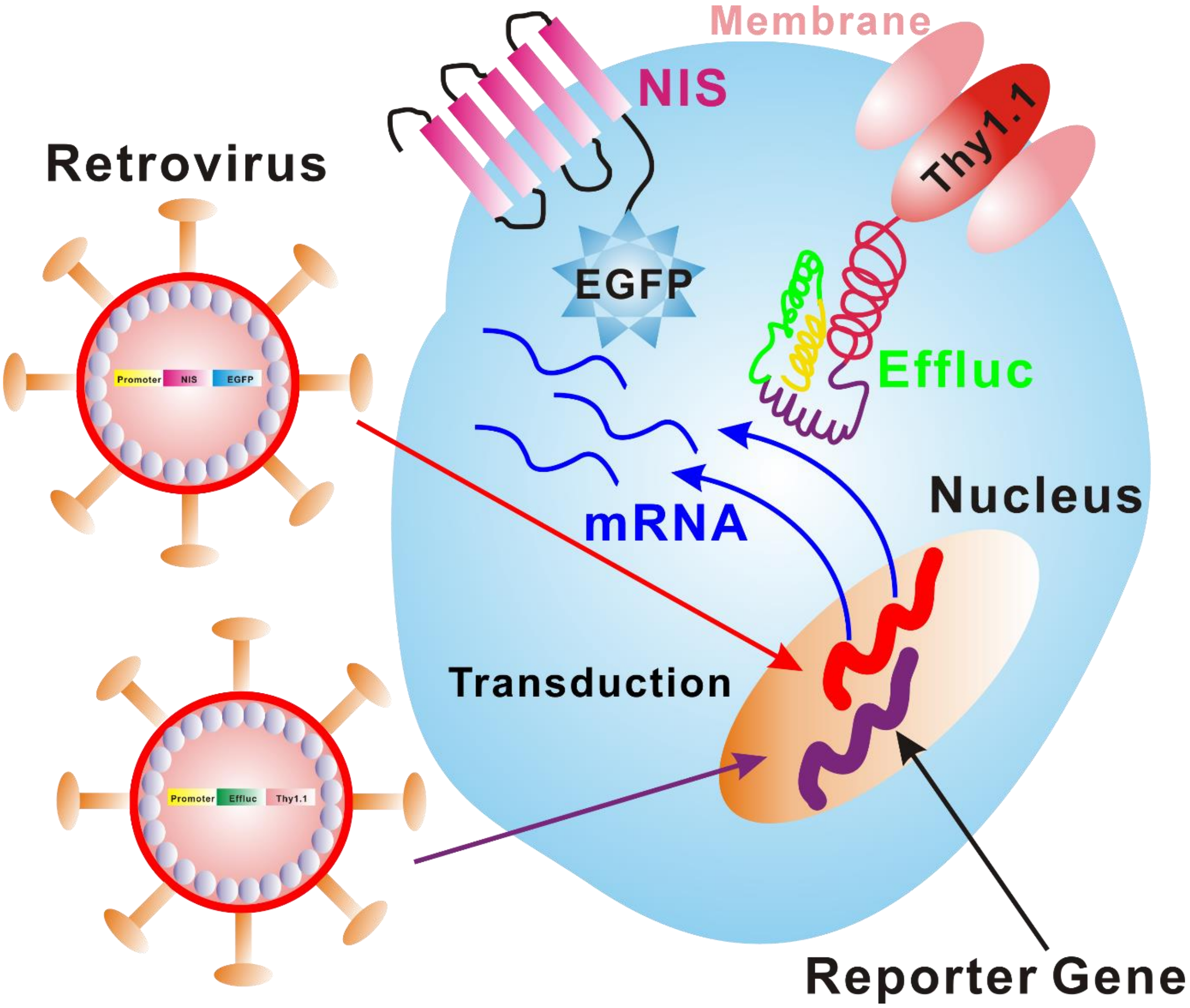

Supplementary Figure 2 Experimental procedures for monitoring the tumor protection effect by E7-transfected DC/NF cells. Briefly, mice received respective DCs by intramuscular injection once a week for 2 weeks, and the mice were challenged with TC-1/RM cells one week after the last vaccination. BLI for the Rluc gene was performed to monitor the tumor growth.

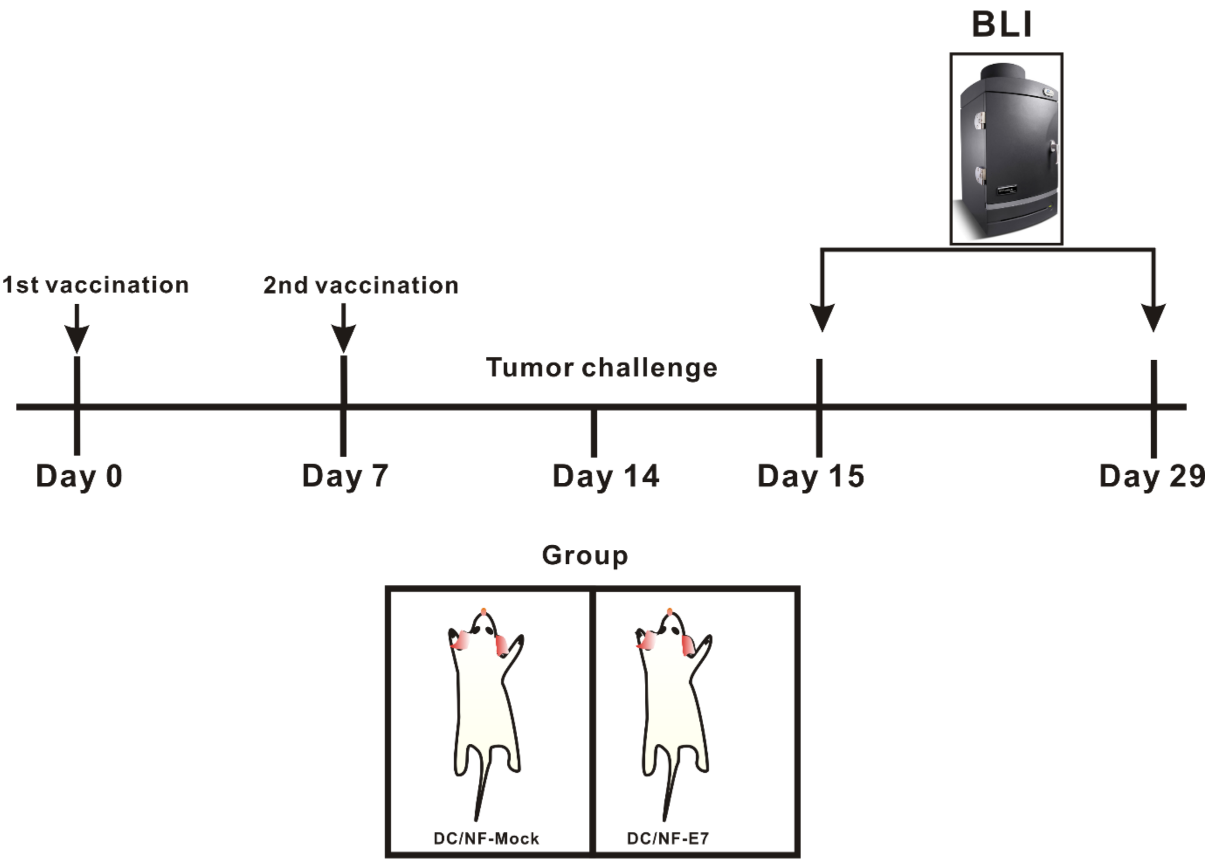

Supplementary Figure 3 Establishment of murine cervical cancer cells expressing Renilla luciferase and mCherry genes and transfection of the E7 gene into DC/NF cells. (A) Luciferase assay for the Rluc gene in TC-1/RM cells. TC-1 cells were transduced with lentivirus co-expressing the Rluc and mCherry genes, and stable clones were enriched by sorting the mCherry-positive population. **(B)** RT-PCR analysis to determine the expression of the E7 gene in DC2.4/NF cells transfected with either pcDNA/mock or pcDNA/E7. GAPDH was used as an internal control gene. Data are expressed as the mean ± SD of 3 independent experiments.

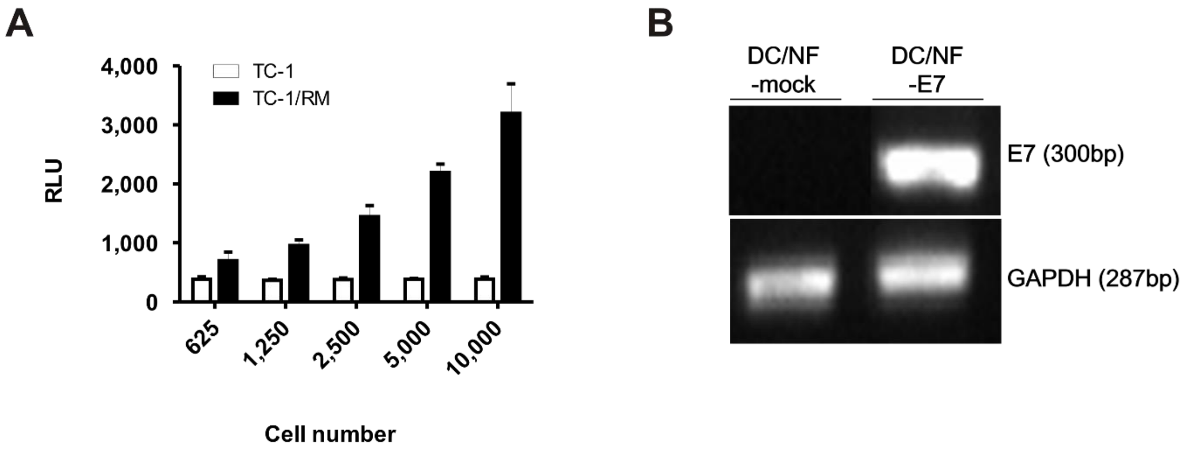

Supplementary Figure 4 Schematic diagram for monitoring DC2.4/NF migration towards the lymph node. Briefly, BLI and I-124 PET/CT imaging were performed on days 1 and 4 after either DC2.4 or DC2.4/NF injection in the right or left hind paw of mice, respectively. After imaging, the respective DPLNs were excised, and both *ex vivo* BLI and autoradiography were performed.

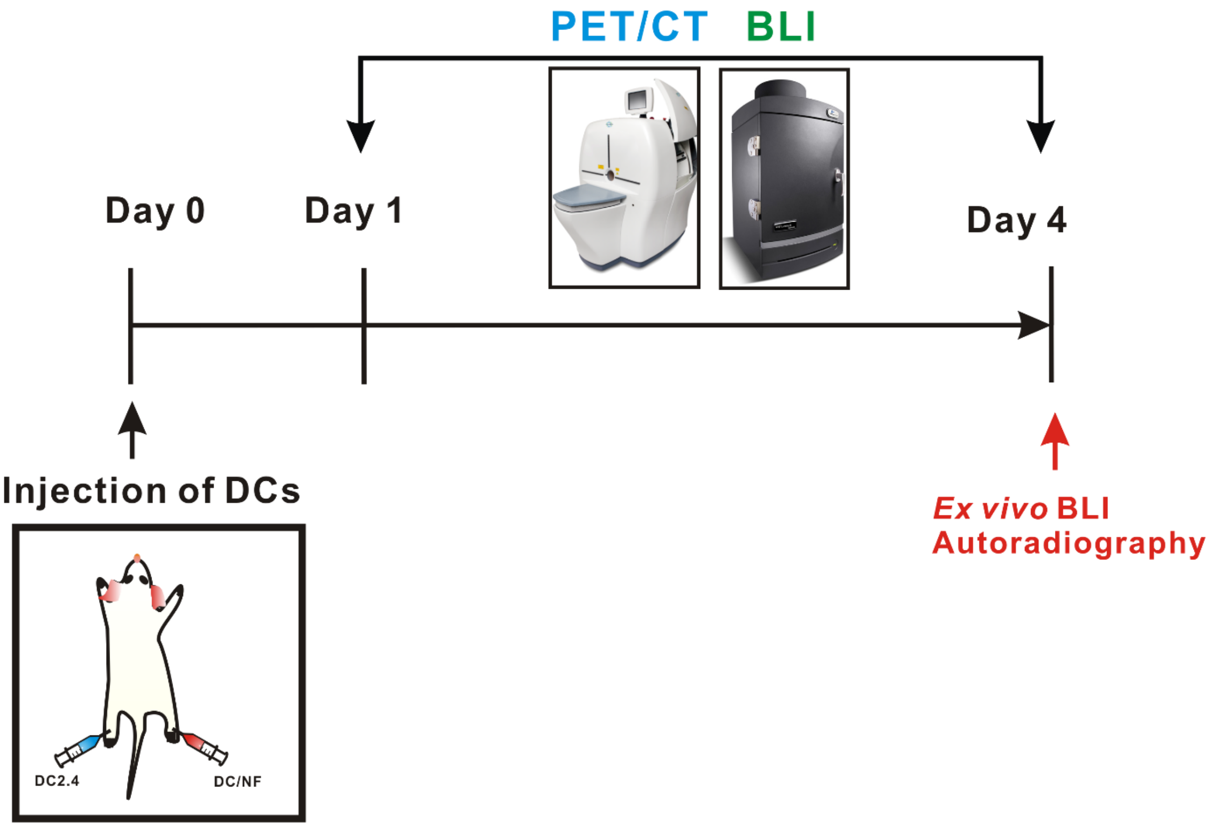

Supplement: Supplementary Information [file srep09865-s1.pdf]
